# Risk factors for hospitalization of patients with chikungunya virus infection at sentinel hospitals in Puerto Rico

**DOI:** 10.1371/journal.pntd.0007084

**Published:** 2019-01-14

**Authors:** Christopher H. Hsu, Fabiola Cruz-Lopez, Danulka Vargas Torres, Janice Perez-Padilla, Olga D. Lorenzi, Aidsa Rivera, J. Erin Staples, Esteban Lugo, Jorge Munoz-Jordan, Marc Fischer, Carlos Garcia Gubern, Brenda Rivera Garcia, Luisa Alvarado, Tyler M. Sharp

**Affiliations:** 1 Centers for Disease Control and Prevention, Poxvirus and Rabies Branch, Atlanta, GA, United States of America; 2 Centers for Disease Control and Prevention, Epidemic Intelligence Service, Atlanta, GA, United States of America; 3 Centers for Disease Control and Prevention, Dengue Branch, San Juan, Puerto Rico; 4 Ponce Health Sciences University, Ponce, Puerto Rico; 5 San Lucas Episcopal Hospital, Ponce, Puerto Rico; 6 Centers for Disease Control and Prevention, Arboviral Diseases Branch, Fort Collins, CO, United States of America; 7 Puerto Rico Department of Health, San Juan, Puerto Rico; University of Texas Medical Branch, UNITED STATES

## Abstract

**Background:**

Hospitalization of patients during outbreaks of chikungunya virus has been reported to be uncommon (0.5–8.7%), but more frequent among infants and the elderly. CHIKV was first detected in Puerto Rico in May 2014. We enrolled patients with acute febrile illness (AFI) presenting to two hospital emergency departments in Puerto Rico and tested them for CHIKV infection to describe the frequency of detection of CHIKV-infected patients, identify risk factors for hospitalization, and describe patients with severe manifestations.

**Methodology/Principal findings:**

Serum specimens were collected from patients with AFI and tested by rRT-PCR. During May–December 2014, a total of 3,035 patients were enrolled, and 1,469 (48.4%) had CHIKV infection. A total of 157 (10.7%) CHIKV-infected patients were hospitalized, six (0.4%) were admitted to the intensive care unit, and two died (0.1%). Common symptoms among all CHIKV-infected patients were arthralgia (82.6%), lethargy (80.6%), and myalgia (80.5%). Compared to patients aged 1–69 years (7.3%), infant (67.2%) and elderly (17.3%) patients were nine and two times more likely to be hospitalized, respectively (relative risk [RR] and 95% confidence interval [CI] = 9.16 [7.05–11.90] and 2.36 [1.54–3.62]). Multiple symptoms of AFI were associated with decreased risk of hospitalization, including arthralgia (RR = 0.31 [0.23–0.41]) and myalgia (RR = 0.29 [0.22–0.39]). Respiratory symptoms were associated with increased risk of hospitalization, including rhinorrhea (RR = 1.68 [1.24–2.27) and cough (RR = 1.77 [1.31–2.39]). Manifestations present among <5% of patients but associated with patient hospitalization included cyanosis (RR = 2.20 [1.17–4.12) and seizures (RR = 3.23 [1.80–5.81).

**Discussion:**

Among this cohort of CHIKV-infected patients, hospitalization was uncommon, admission to the ICU was infrequent, and death was rare. Risk of hospitalization was higher in patients with symptoms of respiratory illness and other manifestations that may not have been the result of CHIKV infection.

## Author summary

Chikungunya is an emerging infectious disease caused by a virus (chikungunya virus, CHIKV) transmitted through the bite of infected mosquitos; typical symptoms are fever and joint pain. After CHIKV was first detected in Puerto Rico in 2014, an epidemic quickly spread across the island. Because previous reports identified varying frequencies of hospitalization of CHIKV-infected patients, we used an existing hospital-based disease detection system to better understand the frequency and reasons for hospitalization of CHIKV-infected patients in Puerto Rico. Among 1,469 patients with laboratory-confirmed CHIKV infection, 11% were hospitalized, most of whom were infants or elderly. Six CHIKV-infected patients were admitted to the intensive care unit, and two died. Although several illness characteristics were associated with hospitalization, most of these were not typical of chikungunya and instead suggested underlying or concomitant respiratory disease. By enrolling patients when they presented to the emergency department and testing them for evidence of CHIKV infection, we determined that hospitalization in this population occurred in roughly one-in-ten CHIKV-infected patients, one-in-two hundred were admitted to the intensive care unit, and one-in-one thousand died. These findings provide information on the spectrum of disease caused by CHIKV, and identified underlying or concomitant respiratory illness as a risk factor associated with hospitalization.

## Introduction

Chikungunya is an acute febrile illness (AFI) characterized by potentially debilitating arthralgia [1] and is the result of infection with chikungunya virus (CHIKV), which is transmitted to humans primarily through the bite of infected *Aedes (Stegomyia)* species mosquitoes [2]. Previous reports have documented explosive outbreaks caused by CHIKV, in which 38–63% of immunologically naive populations were affected [3–8]. Circulation of CHIKV was first reported in the Americas in December 2013 when a patient infected with CHIKV who reported no recent history of travel was identified from the Caribbean island of St. Martin [9]. Outbreaks in other regions of the Caribbean and Americas soon followed, and by the end of 2014 more than 1.2 million suspected chikungunya cases had been reported to the Pan-American Health Organization [10]. Cases continued to be reported from throughout the Americas thereafter, and by the end of 2017 more than 2.6 million suspected cases had been reported [11].

In the United States Caribbean territory of Puerto Rico, the first locally-acquired chikungunya case had illness onset in early May 2014 [12]. Peak incidence of suspected chikungunya cases reported to the Puerto Rico Department of Health (PRDH) occurred in September when more than 4,000 suspected cases were reported per week [13]. In the first year of CHIKV circulation, 28,327 suspected chikungunya cases (8.0 cases per 1000 population) had been reported, and 68% of specimens from suspected CHIKV-infected patients who were tested for CHIKV infection were positive [13]. Testing of specimens collected from asymptomatic blood donors and residents of municipalities in southeastern Puerto Rico demonstrated CHIKV infection rates of 23% and 52%, respectively [14–16], similar to rates seen in other Caribbean islands [17, 18]. Therefore, the actual incidence of CHIKV infection among residents of Puerto Rico was likely larger than that reflected by passive surveillance data.

Previous reports showed that the majority of patients with chikungunya experience an AFI that typically resolves within two weeks [7, 19–22], and can appear clinically similar to influenza, dengue, leptospirosis, and other illnesses that are present throughout the tropics. Though uncommon, frequency of hospitalization of CHIKV-infected patients ranges from 0.5–8.7% [21, 23], and may be affected by access to and timing of seeking medical care, local clinical practices, reporting and testing practices, and frequency of co-morbidities and co-infection. Individuals aged <1 and >65 years and those with co-morbid conditions (e.g., diabetes, cardiovascular disease) have been reported to have increased risk of developing severe manifestations of CHIKV infection [9, 24–28]. Moreover, severe manifestations of CHIKV infection such as meningoencephalitis, bullous skin lesions, and multi-organ failure with hemorrhage were reported during a large outbreak that occurred on Reunion Island in 2006 [29, 30], and sepsis has been reported among CHIKV-infected patients in the Americas [23, 27, 31, 32]. Assessing the frequency of hospitalization and severe manifestations associated with acute CHIKV infection has been challenging due to inconsistent clinical identification and diagnostic testing of patients with suspected chikungunya. Nonetheless, such clinical and epidemiologic characteristics are needed to better understand the natural history of CHIKV infection, inform clinicians of the expected frequency of complications associated with CHIKV infection, and update clinical management guidelines.

Previously reported data from two sentinel AFI surveillance sites in Puerto Rico [13, 33] were analyzed to: 1) determine the frequency of hospitalization of AFI patients with CHIKV infection; 2) compare hospitalized and non-hospitalized CHIKV-infected patients to identify risk factors associated with hospitalization; and 3) describe patients with potentially life-threatening or severe manifestations associated with CHIKV infection.

## Materials and methods

### Ethics statement

Informed consent was provided for all enrolled patients. The Sentinel Enhanced Dengue Surveillance System (SEDSS) study protocol was approved by IRBs at Ponce Medical School Foundation and the U.S Centers for Disease Control and Prevention (CDC). A protocol describing supplemental data collection to identify risk factors associated with hospitalization of CHIKV-infected patients and describe manifestations of severe disease underwent institutional review at CDC and was determined to be public health practice and not research; as such, Institutional Review Board approval was not required for supplemental data collection.

### Study population

This investigation utilized a retrospective, cross-sectional selection of patients who presented for care to either of two hospital emergency departments (ED) in Puerto Rico during May–December 2014 and tested positive for CHIKV infection. San Lucas Episcopal Hospital (SLEH)-Ponce is a tertiary care hospital that receives ~55,000 patients per year and serves as the regional pediatric referral hospital for the Ponce Health District and pediatric referral hospital for the Mayaguez Health District (~500,000 residents). SLEH-Guayama is a secondary care hospital that receives ~6,000 patients per year from southeastern Puerto Rico (~50,000 residents). These sites are two of 62 hospitals that were in operation in Puerto Rico in 2014.

### Data sources

Patients presenting to the hospital ED were queried if they have fever at the moment or have had fever in the past seven days, and those responding in the affirmative (“AFI patients”) were offered enrollment in SEDSS. For those that accepted (“SEDSS participants”), blood, urine, and oronasopharyngeal specimens were collected, and diagnostic testing was performed for dengue, influenza, and other common AFIs. Chikungunya diagnostic testing was added to the SEDSS diagnostic algorithm in April 2014. Demographic characteristics, history of exposures, and clinical data were collected from SEDSS participants during enrollment in the ED.

SEDSS data were supplemented by abstraction of hospitalized case-patients’ medical records to collect data on co-morbid conditions, admitting diagnoses, severe manifestations of CHIKV infection, laboratory results, clinical management and medical interventions, and details of patient outcome. Information not found in the medical records was considered absent.

### Diagnostic methods

Serum specimens collected ≤ 5 days after illness onset were tested by real time RT-PCR, and those collected ≥ 4 days after illness onset were tested by anti-CHIKV IgM antibody capture (MAC) ELISA [34, 35]. Testing for other pathogens that cause AFI were performed as previously described [33].

### Definitions

Because SEDSS participants who tested positive by CHIKV IgM ELISA only were significantly different from those that tested positive by RT-PCR in several relevant characteristics (e.g., age, day post-illness onset of presentation, white blood cell count, frequency of arthralgia), patients who tested positive by IgM ELISA only (n = 97) were excluded from analysis. Hence, “CHIKV-infected patients” were defined solely by detection of CHIKV nucleic acid in a serum specimen by RT-PCR. “CHIKV-negative patients” were defined by testing negative for CHIKV infection by RT-PCR and/or IgM ELISA. A patient with “severe manifestations” was defined as a CHIKV-infected patient who was admitted to the intensive care unit (ICU) or died.

SEDSS and medical chart abstraction data were entered into independent Research Electronic Data Capture (REDCap) databases, and relevant variables were extracted and merged.

### Data analysis

We calculated the frequency of hospitalization among SEDSS patients with confirmed CHIKV infection and among those testing negative for CHIKV infection, and estimated the frequencies of hospitalization attributable to CHIKV infection as: (Frequency of hospitalization among CHIKV-positive patients)–(Frequency of hospitalization among CHIKV-negative patients). We compared the presence or absence of selected characteristics among hospitalized patients to identify risk factors associated with hospitalization.

Chi-squared test and Mood’s median test were used to compare continuous variables. Fisher’s exact test was used for comparison of continuous variables with cell size less than or equal to five. Statistical significance was defined by a p-value < 0.05. Relative risk ratios with 95% confidence intervals were calculated to compare frequencies between dichotomous variables. For dichotomous variables, reference groups were the absence of the indicated variable. Because no significant differences in frequencies of hospitalization by age group were observed between CHIKV-infected patients aged 1–19, 20–49, and 50–69 years, the comparator age group for CHIKV-infected patients aged <1 year or ≥ 70 years was those aged 1–69 years. All analyses were conducted using IBM SPSS Statistical Program 20^th^ version (Armonk, NY).

## Results

### Demographic, clinical, and epidemiologic characteristics of CHIKV-infected patients

**Fig 1:**
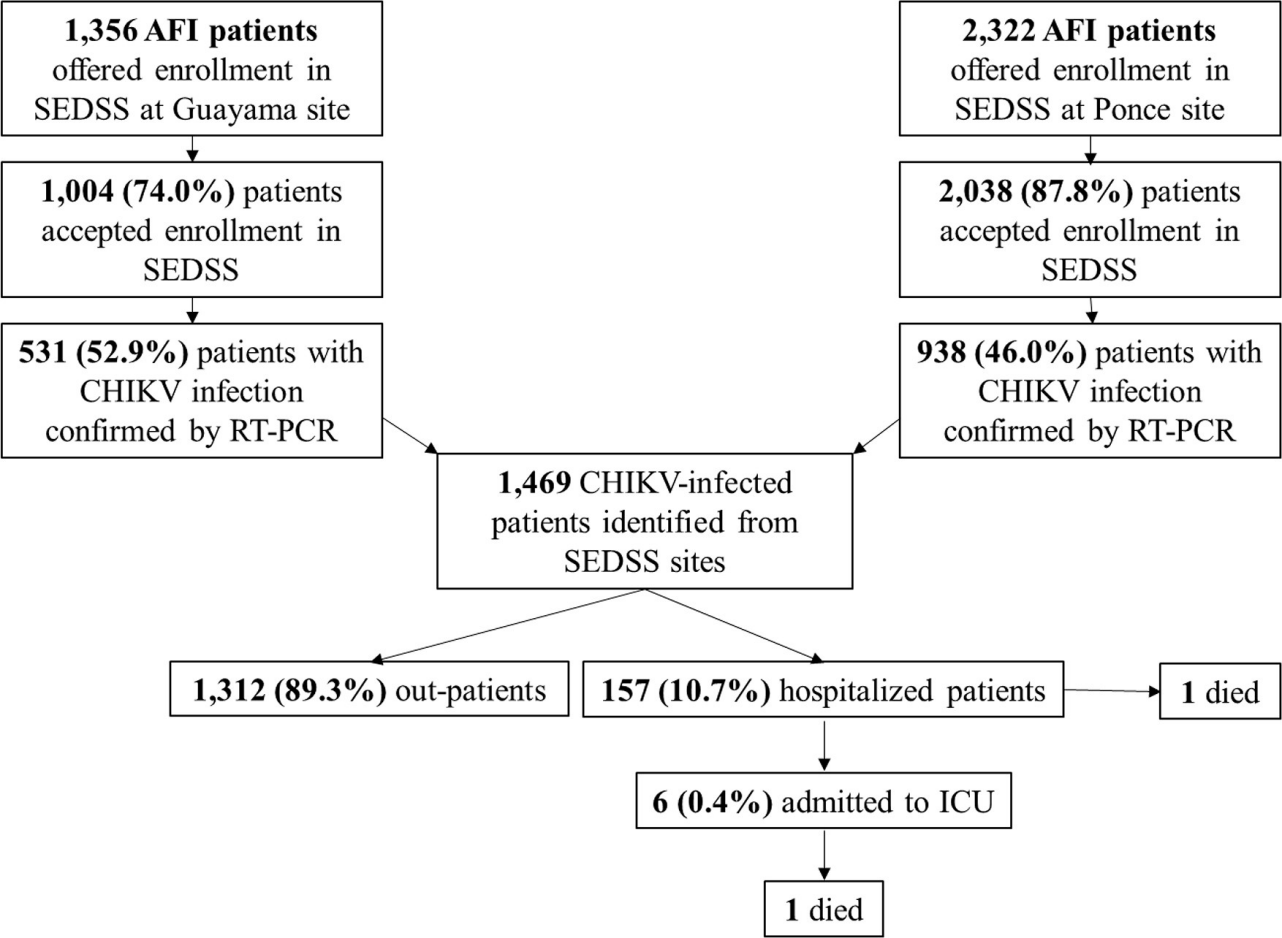
Identification and outcome of chikungunya virus-infected patients enrolled in the Sentinel Enhanced Dengue Surveillance System, May–December 2014.

During May–December 2014, a total of 3,671 AFI patients presenting to SLEH-Ponce and SLEH-Guayama were offered enrollment in SEDSS, of whom 3,035 (82.7%) accepted (Fig 1). The frequency of patients who accepted participation was higher at SLEH-Ponce than at SLEH-Guayama (87.8% vs. 74.0%, respectively; p < 0.01). A total of 1,469 (48.4%) SEDSS participants tested positive for CHIKV infection by rRT-PCR, and were defined as a CHIKV-infected patient. The frequency of SEDSS participants identified as a CHIKV-infected patient was lower at SLEH-Ponce than at SLEH-Guayama (46.0% vs. 52.9%, respectively; p < 0.01). A total of 157 (10.7%) CHIKV-infected patients were hospitalized, of whom six (0.4%) were admitted to the ICU and an additional two (0.1%) died. CHIKV-infected patients were hospitalized more frequently at SLEH-Ponce than SLEH-Guayama (13.4% vs. 7.2%, respectively; p < 0.01). All patients admitted to the ICU and both fatal cases were enrolled at SLEH-Ponce. No CHIKV-infected patients were transferred to another hospital or left the hospital against medical advice.

**Fig 2:**
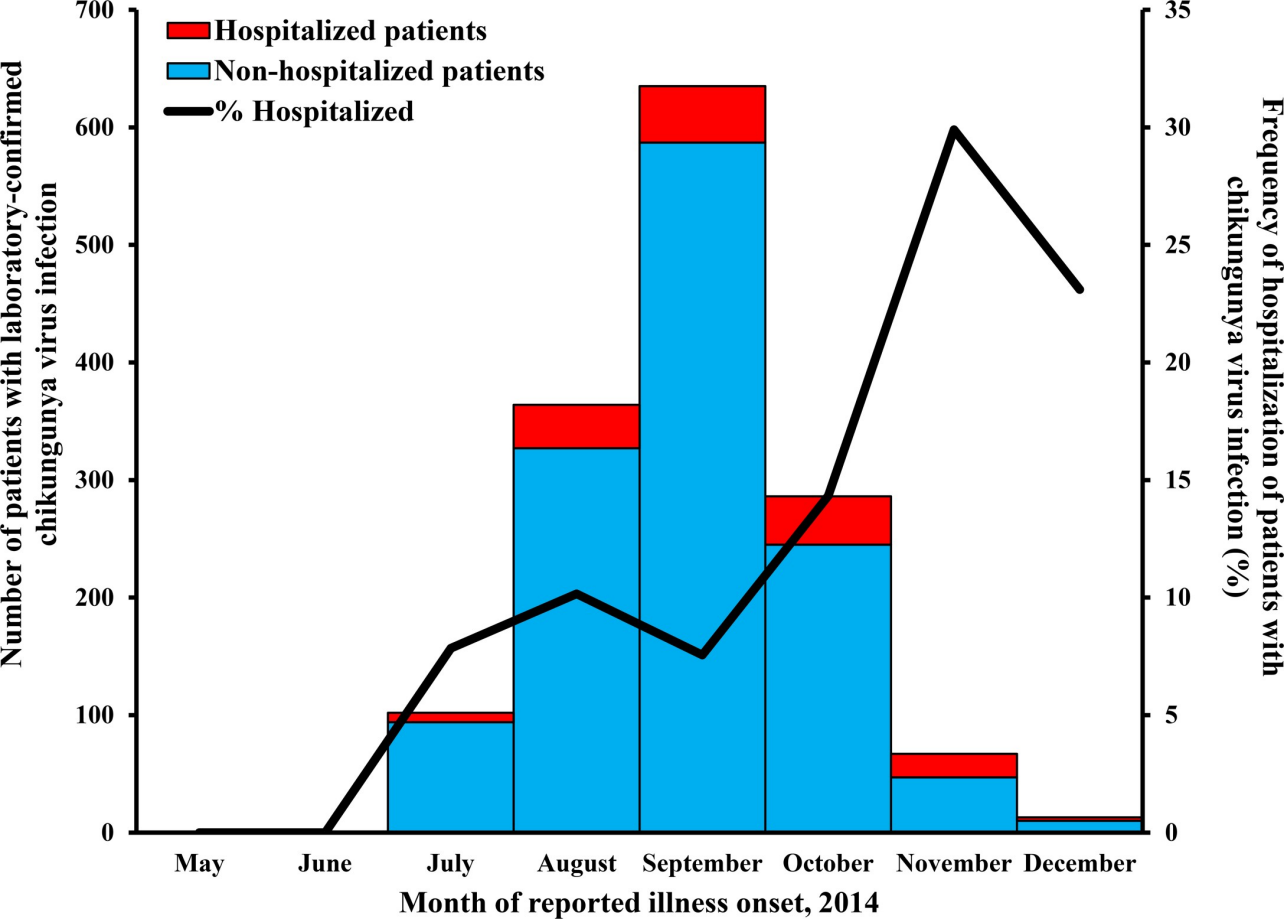
Hospitalized and non-hospitalized chikungunya virus-infected patients enrolled in the Sentinel Enhanced Dengue Surveillance System and frequency of hospitalization by month of illness onset, May–December, 2014.

The first CHIKV-infected patient identified by SEDSS reported illness onset in May 2014 (Fig 2). The number of CHIKV-infected patients progressively increased thereafter, until the peak number of identified patients per month occurred in September (n = 635). The number of detected chikungunya cases steadily decreased thereafter through the end of the year. The monthly frequency of hospitalization of chikungunya cases was relatively consistent throughout the first half of the outbreak (7–10%), but increased in the second half with a peak of 30% in November when only 67 CHIKV-infected patients were identified.

**Fig 3:**
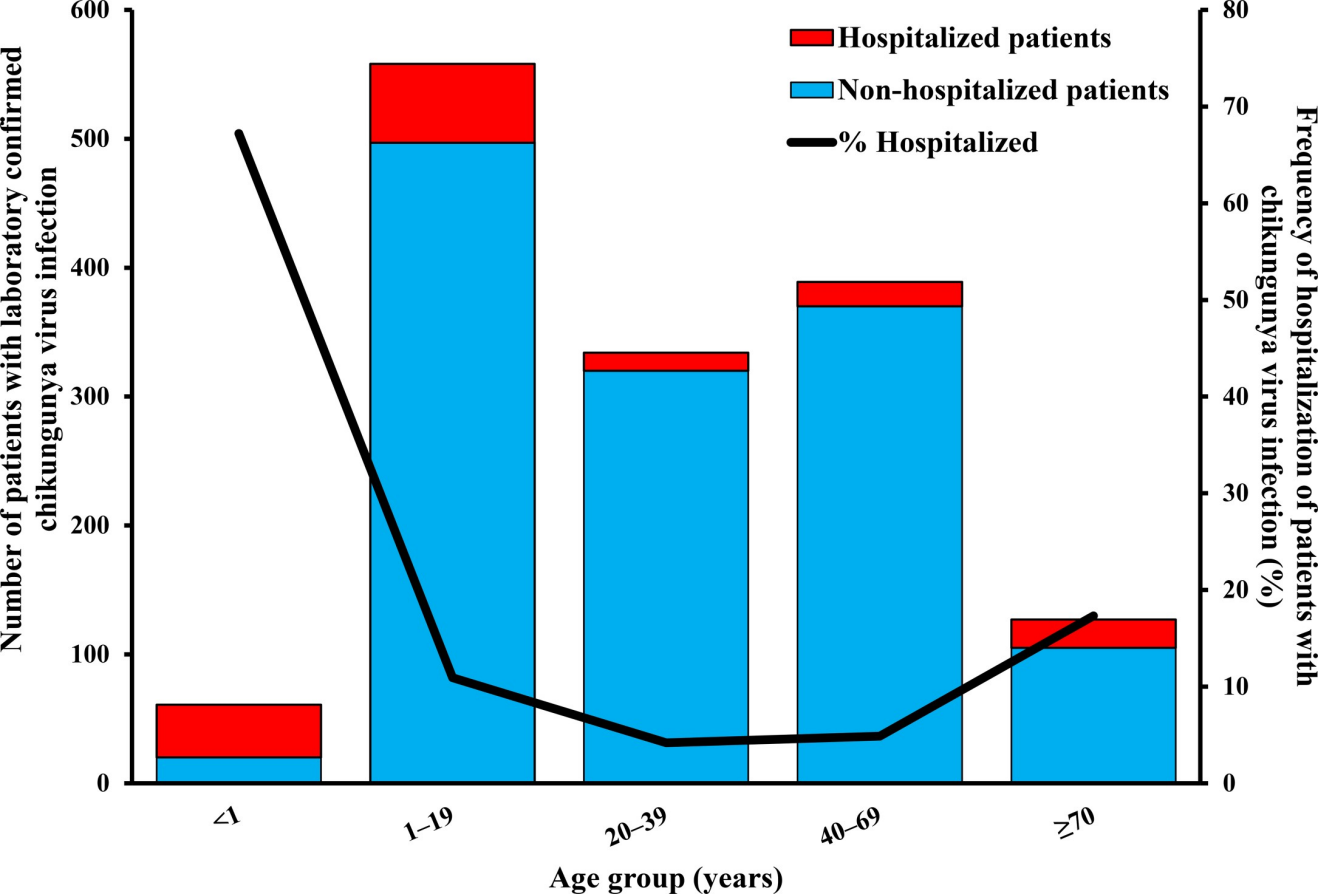
Number and frequency of hospitalization by age group of chikungunya virus-infected patients enrolled in the Sentinel Enhanced Dengue Surveillance System, May–December, 2014.

The greatest proportion of chikungunya cases was among children aged 1–19 years (n = 558; 38.0%) followed by adults aged 40–69 years (389 cases; 26.5%) (Fig 3). Hospitalized patients were significantly younger than non-hospitalized patients (median = 10 vs. 26 years, respectively; p < 0.01). By age group, frequency of hospitalization was highest among CHIKV-infected patients aged < 1 year (67.2%), followed by patients aged ≥ 70 years (17.3%). Hospitalization of CHIKV-infected patients from all other age groups was uncommon (i.e., 4–11%), and did not differ significantly by age group. Day post-illness onset of presentation for care was not significantly different between hospitalized and non-hospitalized patients (median = 1 [range: 0–6] vs. 1 [range: 0–5], respectively; p = 0.74).

Compared to CHIKV-negative patients enrolled in SEDSS during the same period of time, excess frequencies of hospitalization occurred only among infants infected with CHIKV (Table 1). Infants aged <3 months were hospitalized most frequently due to clinical sepsis (11 of 38; 29%), whereas those aged 3–12 months were more frequently hospitalized due to suspicion of chikungunya or viral syndrome (35 of 95; 37%). CHIKV-infected patients of all other age groups were hospitalized less frequently than patients with a non-CHIKV cause of AFI, suggesting that hospitalization of these patients was not attributable to CHIKV infection.

**Table 1:** Crude frequency of hospitalization attributable to chikungunya virus infection among patients with acute febrile illness enrolled in the Sentinel Enhanced Dengue Surveillance System, 2014.

Age group	CHIKV lab-positive			CHIKV lab-negative			Crude frequency of hospitalization attributable to CHIKV infection
	Patients enrolled in SEDSS, N	Patients hospitalized, n	Frequency of hospitalization	Patients enrolled in SEDSS, N	Patients hospitalized, n	Frequency of hospitalization	
<90 days	14	14	100.0%	29	23	79.3%	20.7%
3–<6 months	20	16	80.0%	54	25	46.3%	33.7%
6–<9 months	14	6	42.9%	72	18	25.0%	17.9%
9–<12 months	13	5	38.5%	67	20	29.9%	8.6%
<1 year	61	41	67.2%	222	86	38.7%	28.5%
1–9 years	297	37	12.5%	869	197	22.7%	-10.2%
10–19 years	261	24	9.2%	332	71	21.4%	-12.2%
20–49 years	466	19	4.1%	393	74	18.8%	-14.7%
50–69 years	257	14	5.4%	142	46	32.4%	-27.0%
≥70 years	127	22^†^	17.3%	78	40*	51.3%	-34.0%
All	1,469	157	10.7%	2,036	514	25.2%	-14.5%

*including one fatal case

^†^including two fatal cases

Abbreviations: CHIKV = chikungunya virus; SEDSS = Sentinel Enhanced Dengue Surveillance System

Note: Attributable frequency of hospitalization estimated as (Frequency of hospitalization among CHIKV lab-positive patients)–(Frequency of hospitalization among CHIKV lab-negative patients)

### Characteristics associated with hospitalization of CHIKV-infected patients

Infant and elderly patients were hospitalized approximately nine and two times more often than CHIKV-infected patients aged 1–69 years (Table 2). Patient sex, reported recent history of travel, and reported co-morbid conditions were not significantly associated with hospitalization of CHIKV-infected patients.

**Table 2:** Demographic characteristics, travel history, and co-morbidities among hospitalized and non-hospitalized chikungunya virus-infected patients enrolled in the sentinel enhanced dengue surveillance system, May–December, 2014 (N = 1,469).

	All patients N = 1,469 n (column %)	Hospitalized N = 157 n (row %)	Relative Risk* (95% confidence interval)
**Age group**			
< 1 year	61 (4.2)	41 (67.2)	**9.16 (7.05–11.90)**
1–69 years	1,281 (87.2)	94 (7.3)	Reference
≥ 70 years	127 (8.6)	22 (17.3)	**2.36 (1.54–3.62)**
**Sex**, male	698 (47.5)	84 (12.0)	1.29 (0.96–1.73)
**Traveled to another country in two weeks before seeking care**	19 (1.3)	4 (21.1)	2.00 (0.82–4.83)
High blood pressure	269 (18.3)	28 (10.4)	0.97 (0.66–1.43)
Diabetes	156 (10.6)	22 (14.1)	1.37 (0.90–2.09)
Asthma	218 (14.8)	17 (7.8)	0.70 (0.43–1.13)
Coronary heart disease	85 (5.8)	12 (14.1)	1.35 (0.78–2.33)
Thyroid disease	87 (5.9)	13 (14.9)	1.43 (0.85–2.42)
High cholesterol	109 (7.4)	7 (6.4)	0.62 (0.30–1.29)
Cancer	21 (1.4)	4 (19.0)	1.80 (0.74–4.41)
Chronic kidney disease	21 (1.4)	3 (14.3)	1.34 (0.47–3.87)
Immunodeficiency	12 (0.8)	2 (16.7)	1.57 (0.44–5.60)
Chronic liver disease	8 (0.5)	1 (12.5)	1.17 (0.19–7.37)
Chronic obstructive pulmonary disease	7 (0.5)	0 (0)	0.58 (0.04–8.53)

*For dichotomous variables, reference groups were the absence of the indicated variable.

The most common clinical signs and symptoms of all CHIKV-infected patients when evaluated at the emergency department were arthralgia (82.6%), lethargy (80.6%), myalgia (80.5%), bone pain (78.8%), headache (71.5%), chills (70.7%), rash (61.5%), and conjunctivitis (57.8%) (Table 3). Cyanosis, bleeding gums, hematemesis, hematuria, melena/hematochezia, and seizures were reported in <5% of patients. Clinical signs and symptoms significantly associated with increased risk of hospitalization among CHIKV-infected patients were skin rash, bruises, cyanosis, rhinorrhea, cough, petechia, and seizures. Arthralgia, myalgia, bone pain, calf pain, eye pain, headache, and having pruritic skin were associated with decreased risk of hospitalization among CHIKV-infected patients.

**Table 3:** Signs and symptoms identified during evaluation at the emergency department of hospitalized and non-hospitalized, laboratory-positive chikungunya virus-infected patients enrolled in the sentinel enhanced dengue surveillance system, May–December, 2014 (N = 1,469).

**Signs and symptoms**	**All patients** **(N = 1,469)** **n (column %)**	**Hospitalized** **(N = 157)** **n (row %)**	Relative Risk* (95% confidence interval)
Arthralgia	1,213 (82.6)	93 (7.7)	**0.31 (0.23–0.41)**
Arthritis	642 (43.7)	58 (9.0)	0.75 (0.56–1.03)
Myalgia	1,182 (80.5)	86 (7.3)	**0.29 (0.22–0.39)**
Bone pain	1,157 (78.8)	84 (7.3)	**0.31 (0.23–0.41)**
Calf pain	782 (53.2)	64 (8.2)	**0.60 (0.45–0.82)**
Eye pain	677 (46.1)	48 (7.1)	**0.52 (0.37–0.71)**
Headache	1,050 (71.5)	75 (7.1)	**0.37 (0.27–0.49)**
Chills	1,039 (70.7)	110 (10.6)	0.97 (0.70–1.34)
Lethargy	1,184 (80.6)	129 (10.9)	1.11 (0.75–1.63)
Irritability	436 (29.7)	55 (12.5)	1.28 (0.94–1.74)
Skin rash	904 (61.5)	113 (12.5)	**1.61 (1.15–2.24)**
Pruritic skin	444 (30.2)	32 (7.2)	**0.59 (0.41–0.86)**
Bruises	75 (5.1)	15 (20.0)	**1.96 (1.22–3.17)**
Cyanosis	35 (2.4)	8 (22.9)	**2.20 (1.17–4.12)**
Conjunctivitis	849 (57.8)	90 (10.6)	0.98 (0.73–1.32)
Rhinorrhea	380 (25.9)	58 (15.3)	**1.68 (1.24–2.27)**
Cough	358 (24.4)	57 (15.9)	**1.77 (1.31–2.39)**
Vomiting	202 (13.8)	27 (13.3)	1.30 (0.88–1.92)
Abdominal pain	449 (30.6)	40 (8.9)	0.78 (0.55–1.09)
Diarrhea	253 (17.2)	33 (13.0)	1.28 (0.89–1.83)
Petechia	549 (37.4)	78 (14.2)	**1.65 (1.23–2.22)**
Bleeding gums	35 (2.4)	5 (14.3)	1.35 (0.59–3.01)
Hematemesis	9 (0.6)	2 (22.2)	2.09 (0.61–7.17)
Hematuria	21 (1.4)	2 (9.5)	0.89 (0.24–3.35)
Melena/hematochezia	24 (1.6)	5 (20.8)	1.98 (0.90–4.38)
Seizures	24 (1.6)	8 (33.3)	**3.23 (1.80–5.81)**

*Reference groups were the absence of the indicated variable

Results of routine laboratory tests of blood specimens collected in the emergency department indicated that higher white blood cell count, lower hematocrit, lower platelet count, higher serum creatinine, and higher aspartate amino transferase were more common among CHIKV-infected patients that were hospitalized than among those that were not hospitalized (Table 4).

**Table 4:** Results of laboratory analysis of specimen collected in the emergency department from hospitalized and non-hospitalized, chikungunya virus-infected patients enrolled in the sentinel enhanced dengue surveillance system, May–December, 2014 (N = 1,469).

**Laboratory test**	**Hospitalized** **n = 157**		**Non-hospitalized** **n = 1,312**		**P value**
	Number tested (%)	Value	Number tested (%)	Value	
White blood cell count (x 1,000 per mm^3^), median (range)	157 (100)	8 (2–19)	1,242 (95)	7 (2–22)	<0.01*
<4,000 x 1,000 per mm^3^, n (%)		17 (10.8)		188 (15.1)	0.15
>10,500 x 1,000 per mm^3^, n (%)		32 (20.4)		89 (7.2)	**<0.01**
Hematocrit (%), median (range)	157 (100)	35 (23–48)	1,242 (95)	39 (23–51)	<0.01*
Platelet count (x 1,000 per mm^3^), median (range)	157 (100)	210 (35–635)	1,242 (95)	214 (23–554)	0.69
<100,000 (per mm^3^), n (%)		14 (8.9)		16 (1.2)	**<0.01**
Blood urea nitrogen (mg/dL), median (range)	149 (95)	10 (1–88)	676 (52)	12 (1–71)	0.03*
Creatinine (mg/dL), median (range)	149 (95)	1 (0–18)	676 (52)	1 (0–15)	0.02*
Aspartate amino transferase (units/L), median (range)	107 (68)	36 (11–455)	73 (5.6)	26 (12–719)	<0.01*
Alanine amino transaminase (units/L), median (range)	107 (68)	28 (9–502)	73 (5.6)	23 (12–481)	<0.01*

*Median test or Chi-squared (proportions)

### Description of disease characteristics among hospitalized patients

Throughout the course of hospitalization of 157 chikungunya case-patients, notations in medical records indicated that rash was most often present on the whole body (33%) or localized to either the legs (15%) or trunk (12%), whereas arthralgia was most frequently present in the ankle or knee (12% each), wrist (11%), or hand (10%). Thirteen (8.2%) hospitalized patients had an identified co-infection, many of which were with likely nosocomial pathogens (Table 5). The most common potentially life-threatening manifestations in hospitalized patients were edema and dyspnea; however, edema was most often reported in the ankle (11%), knee (8%), and hands (9%). Vesiculobullous skin lesions were reported in four hospitalized infant CHIKV-infected patients, and were present on the feet in two patients and the hands or mouth in one patient each. Other potentially life-threatening manifestations were infrequently reported, but included: encephalitis in one infant, four adolescents, and one elderly patient; stroke in two adult and two elderly patients; myocarditis in two adults and three elderly patients; and coma in two adolescents and one elderly patient.

**Table 5:** Clinical characteristics of hospitalized chikungunya virus-infected patients enrolled in the Sentinel Enhanced Dengue Surveillance System, May–December, 2014.

**Clinical Manifestations, n (%)**	**All patients** **N = 157**	**Age group**			
		< 1 year n = 41	1–19 years n = 61	20–69 years n = 33	≥ 70 years n = 22
**Arthralgia**	96 (61.1)	9 (22.0)	38 (62.2)	29 (87.9)	20 (90.1)
**Rash**	120 (76.4)	36 (87.8)	53 (86.9)	26 (78.8)	17 (77.3)
**Vesiculobullous lesions**	4 (2.5)	4 (9.8)	0	0	0
**Edema**	39 (24.8)	13 (31.7)	11 (18.0)	4 (12.1)	11 (50.0)
**Dehydration**	69 (43.9)	22 (53.7)	27 (44.2)	10 (30.3)	10 (45.4)
**Dyspnea**	22 (14)	3 (7.3)	6 (9.8)	6 (18.2)	7 (31.2)
Co-infection*	15 (9.6)	3 (7.3)	4 (6.6)	5 (15.2)	3 (12.6)
**Photophobia**	7 (4.5)	0	4 (6.6)	3 (9.1)	0
**Arrhythmia**	11 (7)	0	3 (4.9)	2 (6.0)	6 (27.3)
**Encephalitis**	6 (3.8)	1 (2.4)	4 (6.6)	0	1 (4.5)
**Stroke**	4 (2.5)	0	0	2 (6.0)	2 (9.1)
**Myocarditis**	4 (2.5)	0	0	1 (3.0)	3 (12.6)
**Sepsis**	3 (1.9)	0	1 (1.6)	0	1 (4.5)
**Coma**	2 (1.3)	0	0	2 (6.0)	0
**Neuropathy**	2 (1.3)	1 (2.4)	0	0	0
**Shock**	1 (0.6)	1 (2.4)	0	0	0

*Identified co-infections were: Group B *Streptococcus agalatiae* (3); Respiratory syncytial virus (1); Parainfluenza virus type 3 (1); *Enterococus faecalis* (1); Gram positive cocci (1); Gram positive bacilli (1); Gram positive cocci (1); Unidentified Gram positive bacteria (1); *Staphylococcus aureus* (1); *Enterobacter cloacal* (1); *Pseudomona aeruginosa* (1); *Staphylococcus hominis* (1); *Staphylococcus hominis* (1); and *Enterococcus faecalis* (1).

Seven hospitalized CHIKV-infected patients had severe manifestations associated with CHIKV infection. The first was a 15-month-old female with a history of asthma admitted to the pediatric ICU (PICU) due to chikungunya-like illness and exacerbation of asthma. She was discharged after a three-day stay in the PICU and seven days total in the hospital. The second was a 68-year-old female with petechia, bruising, vesiculobullous skin lesions, and 14,000 platelets per mm^3^ who was diagnosed with immune thrombocytopenic purpura. She was treated with intravenous immunoglobulin and platelet transfusion. Anti-*Leptospira* IgM antibody was detected in blood collected at admission. She was transferred from the ICU after four days and discharged home after a 16-day hospitalization. The third patient was a two-month old male admitted to the PICU due to chikungunya-like illness, edema, suspected sepsis, and pneumonia. He was discharged home after a 13-day hospitalization. The fourth patient was a 29-day-old male who had diffuse rash and bronchial pneumonia, and was hospitalized for 16 days. The fifth patient was a six-day-old male born to a mother with peripartum fever, who was discharged home and re-admitted for fever, rash, respiratory distress, and left hydronephrosis. He was transferred from the PICU on day four, and discharged home the next day.

Two CHIKV-infected patients died during hospitalization. The first was an 80-year-old male with a history of Alzheimer’s disease, dementia, epilepsy, and smoking. He presented with fever, weakness, myalgia, arthralgia, rash, vomiting, and non-bloody diarrhea, and was admitted for dehydration and systemic inflammatory response syndrome. Laboratory values revealed elevated serum troponin (0.100 ng/mL; normal = 0–0.056) suggesting myocardial injury. Blood and urine cultures were negative. Echocardiogram on day four of hospitalization revealed decreased left ventricular ejection fraction, septal hypokinesis, and mitral and tricuspid valve regurgitations. Repeat laboratory values revealed elevated C-reactive protein (13.36 mg/L; normal = 0–0.50), elevated procalcitonin (1.51 μg/L; normal = 0–0.50) indicative of possible systemic infection, and mildly elevated liver enzymes. Chest x-ray on day six of hospitalization revealed left pleural effusion. He exhibited respiratory distress with rhonchi, wheezing, and rales on day 10. Chest x-ray revealed worsening left pleural effusion and bilateral atelectasis. Laboratory results revealed increasing serum troponin (0.385 ng/mL) and elevated CK-MB (4.5 ng/mL; normal = 0–3.6). The following day he was found unresponsive and declared dead. Final diagnoses were aspiration pneumonia, non-ST elevated myocardial infarct, congestive heart failure, respiratory failure, and cardiorespiratory arrest. Autopsy was not requested. No pathogens other than CHIKV were identified.

The second fatal case was a 78-year-old male smoker with a history of hypertension, obesity, and heart and kidney disease. He presented with a one-day history of disorientation, fever, chills, rash, anorexia, myalgia, arthralgia, bone pain, rash, and petechia. He was febrile and had numbness in both lower extremities. Laboratory values revealed mild thrombocytopenia (141,000 cells per mm^3^). He was diagnosed with viral syndrome and acute neurologic deficit and admitted for care. He developed respiratory difficulty and was admitted to the ICU and intubated. Echocardiogram revealed evidence of ST-elevated myocardial infarction, which was consistent with elevated troponin-I (0.070 ng/mL on admission, later elevated to 7.985 ng/mL). He developed progressively worsening bilateral pleural effusions, and the endotracheal tube was observed to be filled with a purulent liquid. His white blood cell count progressed to leukocytosis (17,100 cells per mm^3^) and worsening thrombocytopenia (47,000 cells per mm^3^). On day seven of hospitalization he had an occult hemorrhage requiring transfusion with two units of packed red blood cells. He died on day 10. Cause of death was listed as acute cerebral vascular accident, acute myocardial infarct, respiratory failure, and acute renal failure. A urine culture collected on day three of hospitalization was positive for growth of an unidentified Gram-negative bacilli. Autopsy was not requested.

## Discussion

Following introduction of CHIKV to immunologically naïve populations, chikungunya outbreaks often result in medical services being overwhelmed by patients seeking medical care most often due to debilitating polyarthralgia [36]. Because many CHIKV-infected patients may be diagnosed with an alternative AFI and vice versa, laboratory confirmation is needed to confidently identify patients with CHIKV infection [37–39]. These characteristics together make challenging an accurate estimate of the frequency with which CHIKV-infected patients are hospitalized and develop potentially life-threatening complications. To overcome these challenges, we utilized a previously established facility-based AFI surveillance system in which laboratory-based diagnostic testing was performed on all enrolled patients. This system enabled quantitation of the number of laboratory-confirmed CHIKV-infected patients who presented to the hospital emergency department. Collection of detailed clinical data enabled identification of characteristics in this population that were associated with hospitalization, and description of the frequency with which clinically apparent CHIKV-infected patients had severe manifestations of disease.

The first confirmed case of locally-acquired CHIKV infection in Puerto Rico was detected in the San Juan metropolitan area in early May 2014 [13], and additional cases were detected in Ponce via SEDSS later that month. This timing of the apparent spread of CHIKV is consistent with previous reports of rapid dissemination of CHIKV in naïve populations [40, 41]. The peak number of clinically apparent CHIKV-infected patients detected via SEDSS occurred in September 2014 and declined steadily thereafter, similar to the peak identified by other sources of surveillance for chikungunya cases in Puerto Rico [13, 15]. Thus, the timing of detection of CHIKV-infected patients identified via SEDSS was similar to that observed throughout the island. Moreover, although the temporal trends in CHIKV-infected patient hospitalization were relatively consistent during the eight-month period examined, peak incidence of hospitalization occurred in November when the number of clinically apparent CHIKV-infected patients identified was relatively low. This peak may reflect increased likelihood of hospitalization of CHIKV-infected patients given increases in available hospital resources, patients with milder disease declining to seek clinical care, or increased hospitalization of AFI patients with respiratory signs during influenza season. Nonetheless, hospitalization of 11% of CHIKV-infected patients is consistent with most previous reports [21, 23].

Most hospitalized CHIKV-infected patients identified in this investigation were either from the extremes of age (i.e., infants and the elderly) and/or were hospitalized due to signs and symptoms not consistent with chikungunya. These observations are similar to what has been reported for patients who experienced severe manifestations of disease associated with CHIKV infection during outbreaks in the Americas and elsewhere [2, 23, 27–29, 36, 40]. The highest frequency of hospitalization by age group was among infants, who have been demonstrated to be at higher risk for severe manifestations of disease associated with CHIKV infection, particularly neonates and those infected through perinatal transmission [2, 36, 42]. Interestingly, in contrast to patients of all other age groups, infants with CHIKV infection were hospitalized more frequently than those with a non-CHIKV etiology of AFI, suggesting increased disease severity (e.g., higher fever, irritability). A recent study of hospitalized febrile infants with CHIKV infection found that the highest frequency of signs and symptoms included irritability, rash, and edema, all of which were similar to our findings [42].

Although the extremes of age were significantly associated with increased risk of hospitalization for the patients in this investigation, we did not observe that comorbid conditions were associated with hospitalization. This finding is in contrast to prior studies that associated hospital admission among CHIKV-infected patients with comorbidities such as cardiac disease, chronic renal disease, diabetes, and lung disease [9, 24–28, 43, 44]. However, we note that such observations were often gleaned from case series of CHIKV-infected patients who were hospitalized or had severe manifestations of disease instead of comparing laboratory-confirmed hospitalized and non-hospitalized CHIKV-infected patients. Alternatively, this discrepancy could represent differences in patient admission practices. We also cannot discount a possible effect of one-quarter of all hospitalized patients being infants and therefore unable to declare symptoms, which may have affected the observed associations.

Consistent with the well-described clinical manifestations associated with chikungunya [2, 36, 45], rash, arthralgia, myalgia, headache, and chills were the most common symptoms reported among CHIKV-infected patients. However, because the case definition for patients offered enrollment in SEDSS included fever, we cannot rule out possible manifestations of CHIKV infection in patients that did not report fever. Hospitalized CHIKV-infected patients more often had rash, bruises, cyanosis, rhinorrhea, cough, petechia, and seizures, whereas those discharged from the emergency department more often had classic manifestations of chikungunya. These findings together suggest that patients with uncomplicated chikungunya were more likely to be discharged home, whereas those with manifestations outside of the expected clinical spectrum of disease and more similar to another etiology of AFI (e.g., influenza virus) were more likely to be hospitalized. Such manifestations in hospitalized CHIKV-infected patients are likely to be attributable to either: atypical manifestations of CHIKV infection; co-infection (which we were unable to reliably compare between hospitalized and non-hospitalized patients); or exacerbation of underlying conditions. Previous studies have also reported similar, non-specific clinical manifestations among CHIKV-infected patients, but none have reported consistent anatomical findings or clinical progression [22, 43, 44, 46]. Finally, elevated transaminase levels, leukocytosis, thrombocytopenia, and elevated creatinine have been previously associated with CHIKV infection in adults [43, 44], all of which were observed to be associated with hospitalized patients in this investigation, though not exclusively in adults.

The frequency of severe manifestations associated with CHIKV infection identified in this investigation, as defined by admission to the ICU or death among clinically apparent CHIKV-infected patients, was low (i.e., 0.4%). As previously reported, several such patients had severe underlying co-morbidities, whereas others had manifestations associated with CHIKV infection including encephalitis, sepsis, and vesiculobullous skin lesions [2, 29, 36]. One patient was apparently infected via vertical transmission. Although other hospitalized patients had characteristics of illness previously considered to be severe manifestations of CHIKV infection (e.g., sepsis, encephalitis), not all such patients had illness that was clinically severe enough to necessitate admission to the ICU. One CHIKV-infected patient was diagnosed with and treated for immune thrombocytopenic purpura (ITP), which has not previously been associated with CHIKV infection; however, CHIKV infection has been associated with fatal thrombotic thrombocytopenic purpura [TTP] [47]. The potential relationship in the case reported herein was complicated by serologic evidence of recent infection with *Leptospira* species bacteria, which has been associated with TTP [48], but not ITP.

This investigation also confirms that fatal outcomes rarely occur in association with CHIKV infection (i.e., 0.1% of clinically apparent cases that presented to an emergency department) [2, 27, 29, 36]. Although both fatal cases identified in this investigation had severe manifestations previously associated with CHIKV infection [23, 27, 29], they also had severe underlying comorbidities that likely complicated their clinical course. Consequently, the role of CHIKV infection in these fatal outcomes was unclear. Future efforts to evaluate the role of CHIKV infection in fatal cases should emphasize collection of tissue specimens during autopsy to more confidently assess a potential role for CHIKV in the clinical course and outcome.

A primary strength of this investigation was utilization of consistent methodology for both identifying and performing laboratory diagnostic testing on patients likely to have CHIKV infection. Nonetheless, the findings are subject to several limitations. First, the patient population was limited to two sentinel hospitals in southern Puerto Rico. Because hospital admission practices may differ between hospitals, the observed frequencies of patient hospitalization may not be representative of all health facilities in Puerto Rico. In addition, clinicians’ familiarity in managing dengue patients may have led to hospitalization of patients with CHIKV infection who had clinical findings that are not necessarily associated with poor outcome (e.g., thrombocytopenia). Because of observed clinical and demographic differences between patients who had detectable CHIKV nucleic acid versus anti-CHIKV IgM antibody, our patient population was limited to those in whom CHIKV nucleic acid was detected in blood at the time of presentation. This approach may have resulted in exclusion of true CHIKV-infected patients who presented later in the course of disease and therefore may have had different demographic or clinical characteristics. Last, the generalizability of the findings are limited, as some are dependent upon co-circulation of other pathogens (e.g., respiratory viruses) and the characteristics of the populations that they most affect (e.g., infants and elderly). Prospective studies that account for such differences would more reliably identify factors associated with disease severity.

To our awareness, this investigation represents the largest cohort of prospectively identified, laboratory-confirmed CHIKV-infected patients. The observed trend of increased frequency of hospitalization of infant and elderly CHIKV-infected patients, particularly those with uncommon and potentially severe manifestations associated with CHIKV infection, underscores the challenge of identifying patients infected with CHIKV based solely upon clinical presentation. Diagnoses at the time of discharge of hospitalized and non-hospitalized CHIKV-infected patients included dengue, influenza, and pneumonia, further demonstrating the difficulty of clinical diagnosis of some CHIKV-infected patients. These clinical misdiagnoses demonstrate that accurate and rapid laboratory-based diagnostic testing for CHIKV-infected patients is imperative in areas where the virus has become endemic, including the need for differentiation from patients with alternative etiologies of AFI for which early diagnosis is associated with reductions in morbidity and mortality [49–51].

## Supporting information

S1 Checklist STROBE checklist. (DOC)

S1 File (XLSX)
